# Excess Absorbance as a Novel Approach for Studying the Self-Aggregation of Vital Dyes in Liquid Solution

**DOI:** 10.3390/ijms24021645

**Published:** 2023-01-13

**Authors:** Antonio Minó, Lucio Zeppa, Luigi Ambrosone

**Affiliations:** 1 Department of Biosciences and Territory (DiBT), University of Molise, Contrada Lappone, Pesche, 86090 Isernia, Italy; 2 Corneal Transplant, Department of Medicine, Hospital S. G. Moscati, Contrada Amoretta, 83100 Avellino, Italy; 3 Department of Medicine and Health Sciences “V. Tiberio”, University of Molise, Via F. De Sanctis, 86100 Campobasso, Italy

**Keywords:** excess absorbance, self-aggregation, equilibrium constant

## Abstract

In the present paper, a simple method for analyzing the self-aggregation of dyes in a solution by a UV-visible absorption measurements is proposed. The concept of excess absorbance is introduced to determine an equation whose coefficients determine the parameters of the aggregation equilibrium. The computational peculiarities of the model are first discussed theoretically and then applied to sodium fluorescein in polar protic and aprotic solvents, as well as in aqueous solutions of methylene blue, which is a cationic dye. Although the experimental responses are very different, the model appears to work equally well in both cases. The model reveals that the trimer is the most likely configuration in both solvents. Furthermore, aggregation is strongly favored for the protic solvent. Interestingly, the model establishes that in aqueous solutions of methylene blue, the tetramer is the predominant form, which has long been assumed and recently demonstrated with sophisticated computational techniques.

## 1. Introduction

A dye can generally be defined as a colored chemical compound that possesses an affinity with the substrate to which it is being applied. Organic dyes are widely used in biomedical imaging [1]; biological and chemical sensing [2]; as well as in photoelectrochemical cells [3]. Of particular interest, are the vital dyes used as a diagnostic tool. These allow surgeons to better visualize membranes and semitransparent intraocular tissues, such as the internal limiting membrane [4,5]. Furthermore, they have to be administered in the form of organic or aqueous solutions, such that the problem of the minimum toxicity dose arises [6,7]. Clearly, an excessive decrease in the dye concentration implies a drastic reduction in the residence time of the dye. This is especially the case regarding the specific site, which is a compromise of the surgical intervention. However, in order to establish the optimal performance of a dye, it is necessary to know its physicochemical behavior in a solution and the impact it has on its photophysical properties [8].

The chemical effects in a solution are generally classified in terms of association or solvation. By the former, we mean the dye’s tendency to form “non-covalent” polymers. By solvation, we mean the tendency of dye molecules to form complexes. It is easy to see that whenever solvation occurs in a solution it has a marked effect on the thermodynamic properties of that solution. It is perhaps not so obvious to note the association effects, nor when they occur. However, both are of major importance. The reason for this is that the extent of association is a strong function of the composition, especially in the range that is dilute with respect to the associating component [9]. The ability of a dye to solvate or associate is closely related to its electronic structure, thus various studies have been performed in order to correlate the chemical structure of dyes to the Lambert–Beer law [10]. This law is an empirical tool employed for determining the purity or the concentration of an absorber. Furthermore, it is also applied for the concentration measurements of living tissue chromophores, as well as in the estimation of physiological parameters, e.g., blood oxygen saturation [11,12]. The Lambert–Beer law states that the absorptive capacity of a dissolved substance is directly proportional to its concentration, namely that the chromophore absorbance is a linear function of its concentration. Moreover, it requires less computational power when compared to other approaches, such as diffusion approximation [13], Monte Carlo simulation [14] or artificial neural networks [15].

It is important to note that the association of dyes in a solution has been considered detrimental for its functional properties [16]. In recent years, the scientific community has recognized the benefits of π-conjugated materials [17]. It is not surprising, therefore, that the aggregation state of dyes in a solution has also been investigated by a variety of methods, including conductivity [18], calorimetry [19], polarography [20], self diffusion measurements [21], visible light absorption [22,23] and fluorescence emission [24,25]. Due to the non-linearity of the equations, the extent of association is only rarely calculated and the calculation of the equilibrium constants are limited to the dimerization reaction [23].

In this paper, the Lambert–Beer law is considered to be an *ideal condition* where, at a constant temperature and pressure, the absorbance of every absorber is proportional to its concentration. The real behavior is then related to an idealized model (i.e., the Lambert–Beer law) by the *excess absorbance*. This function may be positive or negative, depending on the specific interactions occurring in the solution. Our aim is to show that for any dye solution—regardless of whether its excess absorbance is positive, negative or how it is generated—thermodynamic properties of interest in aggregation equilibria can be calculated from spectrophotometric measurements via excess absorbance. In particular, a simple mathematical relationship that allows one to extend the equilibrium to a number *n* of the dye molecules that are involved is derived. A numerical procedure for extracting the equilibrium parameters and the aggregation number from the equation is discussed in detail. The theoretical model is then applied to sodium fluorescein in an aqueous solution as an example of positive excess absorbance; to sodium fluorescein in acetonitrile solution as an example of negative excess absorbance and finally to aqueous solutions of methylene blue as an example of cationic dye [26].

These dyes are taken as an example because they are widely used in medicine. Indeed, fluorescein is important in ophthalmology to assess ocular surface integrity and to diagnose and follow-up with patients who possess dry eye syndrome [27], as well as to analyze the appearance and disappearance of microaneurysms in different phases of angiography [28]. Regarding methylene blue, in addition to being a surgical dye, it has recently also been used to understand the aging process [26]. It should also be noted that, although dyes are a valuable aid to the surgeon, their adverse effects cause great concern [29]. We are confident that the proposed method is useful for acquiring physical and chemical information on the dye solutions to aid the surgeon in choosing the type of dye and the minimum concentration to be used in operations.

## 2. Results and Discussion

### 2.1. Theoretical Aspects

#### 2.1.1. Integral Absorbance

The macroscopic description of optical absorption, known as the Lambert–Beer law, is widely used in spectroscopy. It states that the light propagating across an absorbing medium is attenuated at a constant rate [30]. The Lambert–Beer law can also be expressed into the sample as the remaining light intensity at a depth *x*. If the absorbing medium is a dye solution, the light penetration length is proportional to the dye concentration. Furthermore, it takes a particularly simple form, i.e.,

(1) $$A_{exp}=\epsilon \left(\lambda \right)\ell C$$

where $A_{exp}$ is the measured absorbance, *C* is the dye concentration in a molar unit, ϵ(λ) is the absorptivity coefficient and *ℓ* is the length of the path light that must travel in the solution.

Thanks to its simplicity, Equation (1) is a useful and practical tool in many areas of science (for instance, in chemistry) in order to determine the degree of purity of organic compounds or their concentration. For analytical purposes, the concentration is measured in molar units and *ℓ* coincides with the thickness of the measuring cuvette (in cm units). Analyses are generally performed with monitoring the absorption spectra at the wavelength of maximum absorption. However, a single wavelength poorly captures the magnitude of the absorption, as this is the feature that is most directly structure-related and thus predictable. In order to overcome this drawback, we recently proposed to generalize the Lambert–Beer law by introducing the integral absorbance [23,25] in the interval $\left[\lambda_{1},\lambda_{2}\right]$ of the absorption band, i.e.,

(2) $${\bar{A}}_{exp}=\bar{\epsilon }\ell C$$

where ${\bar{A}}_{exp}$ is the area under the absorption band and $\bar{\epsilon }$ is the integral molar absorption coefficient defined as

(3) $$\bar{\epsilon }=\int_{\lambda_{1}}^{\lambda_{2}}\epsilon \left(\lambda \right)d\lambda$$

If there are more absorbent species in the solution, Equation (1) provides the contribution of each chromophore, as well as the experimental spectrum that is the superposition of all contributions as weighted by their concentrations. The aggregation of dyes in a solution is a dynamic phenomenon in which *n* monomeric dye molecules associate to form an aggregate D_*n*_, i.e.,

(4) $$nD⇄D_{n}$$

where D represents 1 mole of a pure monomer dye and D_*n*_ is 1 mole of the aggregate containing *n* dye molecules. This model thus assumes a dissociation–association equilibrium between dye monomers and aggregates, as well as an equilibrium constant, which can be calculated as

(5) $$K_{n}=\frac{C_{n}}{C_{1}^{n}}$$

where $C_{1}$ and $C_{n}$ are the concentrations of the dyes in the monomer and aggregate form, respectively.

The ability of a dye molecule to absorb light is closely related to its electronic structure and the chemical environment in which it is embedded. This implies that a same dye molecule, in the monomer and aggregate form, exhibits two distinct absorption coefficients. Accordingly, the absorbance of a dye solution where an aggregation equilibrium takes place can be written as

(6) $${\bar{A}}_{exp}={\bar{\epsilon }}_{1}\ell C_{1}+n{\bar{\epsilon }}_{n}\ell C_{n}$$

where the individual concentrations are linked by the material balance

(7) $$C=C_{1}+nC_{n}$$

where *C* is the total dye concentration.

#### 2.1.2. Excess Absorbance

Equation (6) expresses the macroscopic theory of light absorption in terms of specific forces of attraction that lead to the formation of new molecular species. In order to numerically relate this macroscopic theory and its chemical forces, it is desirable to establish a connection between the parameters of the theory and the properties of the chemical equilibria via a suitable model. On the other hand, one of the essential requirements for a successful model in the physical sciences is judicious simplification. Indeed, if one wishes to do justice to all the aspects of a problem, one very soon finds oneself in a hopelessly complicated situation and thus, in order to make progress, it is necessary to ignore certain aspects of a physical situation and to retain others. The specific assumptions that form the basis of our model are that: (a) the optical properties with infinite dilution are such due to the interactions with the solvent; (b) there is only one equilibrium throughout the concentration range and (c) the extinction coefficients, ${\bar{\epsilon }}_{1}$ and ${\bar{\epsilon }}_{n}$, are constant throughout the concentration range. Excess absorbance is a spectrophotometric property of solutions that are in excess of those of a Lambert–Beer solution at the same conditions of temperature, pressure and composition. The excess absorbance is defined by:

(8) $$\begin{array}{c} \Omega \left(C\right)={\bar{A}}_{exp}-{\bar{\epsilon }}_{D}C\ell \end{array}$$

where ${\bar{A}}_{exp}$ is the integral absorbance that is experimentally obtained as a function of dye concentration and ${\bar{\epsilon }}_{D}$ is the fictitious integral extinction coefficient, which would be obtained if the Lambert–Beer law were valid in the entire concentration range that was investigated.

The excess absorbance is relative to a fictitious solution where the Lambert–Beer law is always satisfied. Clearly, the numerical value of the excess absorbance provided in Equation (8) depends on how the ${\bar{\epsilon }}_{D}$ is defined. Since there is flexibility with respect to a choice of *reference state*, we thus used the infinitely dilute solution.

On the other hand, the curve ${\bar{A}}_{exp}\left(C\right)$—in the extremely diluted solution region—is a monotonic function, such that its tangent in the origin either lies entirely above the curve (concave curve) or is entirely below it (convex curve). It follows that the excess absorbance exhibits either a minimum or a maximum in the origin, thus making use of the fact that $C_{1}\to 0$ as C→0, which is where infinite dilution occurs, as detailed in

(9) $$\lim_{C\to 0}\frac{d\Omega }{dC}=\lim_{C_{1}\to 0}\frac{d{\bar{A}}_{exp}}{dC_{1}}-{\bar{\epsilon }}_{D}\ell =\left({\bar{\epsilon }}_{1}-{\bar{\epsilon }}_{D}\right)\ell =0$$

When choosing the infinitely diluted solution as a reference, $\epsilon_{D}$ coincides with the extinction coefficient of the monomer. This provides a convenient and useful way by which to calculate excess absorbance. It should be noticed that excess absorbance may be positive or negative; furthermore, when Ω(C) is greater than zero, the experimental curve, ${\bar{A}}_{exp}$, is convex—whereas if Ω(C) is less than zero, the experimental curve is concave. By a substitution of Equations (6) and (7) into Equation (8), the expressions of $C_{1}$ and $C_{n}$ as a function of *C* and Ω are found

(10) $$C_{1}=\frac{\Delta_{n}C-\Omega \left(C\right)}{\Delta_{n}},\;C_{n}=\frac{\Omega (C)}{n\Delta_{n}}$$

Although the Ω(C) function may theoretically assume both positive and negative values, for reasons of numerical stability it is useful to process the data points such that Ω>0. This, therefore, implies that $\Delta_{n}>0$, i.e.,

(11) $$\begin{array}{c} \Delta_{n}=\left({\bar{\epsilon }}_{n}-{\bar{\epsilon }}_{1}\right)\ell \;\mathrm{if}\;{\bar{A}}_{exp}\left(C\right)\;\mathrm{is}\;\mathrm{concave} \end{array}$$

(12) $$\begin{array}{c} \Delta_{n}=\left({\bar{\epsilon }}_{1}-{\bar{\epsilon }}_{n}\right)\ell \;\mathrm{if}\;{\bar{A}}_{exp}\left(C\right)\;\mathrm{is}\;\mathrm{convex} \end{array}$$

Substitution of Equation (10) into Equation (5) results in

(13) $$C=\frac{1}{\Delta_{n}}\Omega +\frac{1}{{\left(nK_{n}\Delta_{n}\right)}^{\frac{1}{n}}}\Omega^{\frac{1}{n}}$$

which represents the key equation of the model. Therefore, it becomes crucial to implement the computational procedure to extract the thermodynamic parameters from this equation.

In order to calculate $\epsilon_{1}$, two methods can be employed. If the experimental curve is smooth, a linear fitting on the first three points provides the slope of the curve at infinite dilution. Conversely, when the curve is rough, one may use the method described elsewhere [31] in order to calculate the partial molar volumes of the solutions. Briefly, the data are first smoothed and then the first five points are fitted with a parabola whose derivative for C=0 gives the $\epsilon_{1}$ value.

#### 2.1.3. Computational Procedure

In order to evaluate ${\bar{\epsilon }}_{1}$, a reliable numerical method must be available. Since Lambert–Beer’s law is valid in the limit of C→0 (see Equation (9)), a linear extrapolation of ${\bar{A}}_{exp}$ against the concentration determines ${\bar{\epsilon }}_{1}\ell$. On the other hand, the parameter *ℓ* is an instrumental characteristic fixed by the specific cuvette used in the experiment, such that the slope at the origin of the curve directly provides $\epsilon_{1}$.

When assigned the value ${\bar{\epsilon }}_{1}$, the set of data points (Ω,C) are fitted to equation

(14) $$C=a\Omega +b\Omega^{\frac{1}{n}}$$

where *a* and *b* are adjustable and n≥2 is the association number that takes integer values.

Thus, one proceeds as follows, for n=2,3,4,⋯n. As such, the data set is fitted with a non negative least square procedure. For each *n*, the accuracy is measured by computing the root mean squared error (RMSE) of the data set when compared to the predicted value. From the plot of the RMSE vs. *n* one determines the minimum that is assumed to be the representative point of the system. Then, the triplet (n,a,b) is used to extract both the aggregation equilibrium constant and the absorbing properties of the aggregate, i.e.,

(15) $$K_{n}=\frac{a}{nb^{n}},\;\Delta_{n}=\frac{1}{a}$$

The procedure described above deserves a few words of comment. Indeed, when applying a least squares method to experimental data, it is usually assumed that the error on the *independent* variable is negligible, such that the error minimized by the procedure is attributable only to the *dependent* variable. While the uncertainty about the size *C* can easily be estimated to be 1–2%, it is difficult to establish the reliability of Ω due to the different mathematical operations. For this reason, we propose to use the parameters $n,K_{n}$ and $\Delta_{n}$, which are established at the minimum RMSE. This is performed in order to calculate the integral absorbance in an alternative manner and to then compare the results with the experimental values. In particular, the parameters *n* and $K_{n}$ extracted at the minimum RMSE are used, together with Equation (7) in order to transform Equation (5) into the following algebraic equation

(16) $$\left(nK_{n}C^{n-1}\right)\xi^{n}+\xi -1=0$$

where $\xi =C_{1}/C$ is the dye fraction in the monomer form. For each concentration value, a solution $\xi_{0}$ of Equation (16) is calculated. Hence, the set of values $\left(\xi_{0},C\right)$ determine $C_{1}$ and $C_{n}$ from which the integral absorbance, ${\bar{A}}_{calc}$, is calculated. Based on the comparison between ${\bar{A}}_{exp}$ and ${\bar{A}}_{calc}$ one is able to establish the reliability of the calculated parameters.

### 2.2. Applications

#### 2.2.1. Anionic Dye in Water Solutions

**Figure 1:**
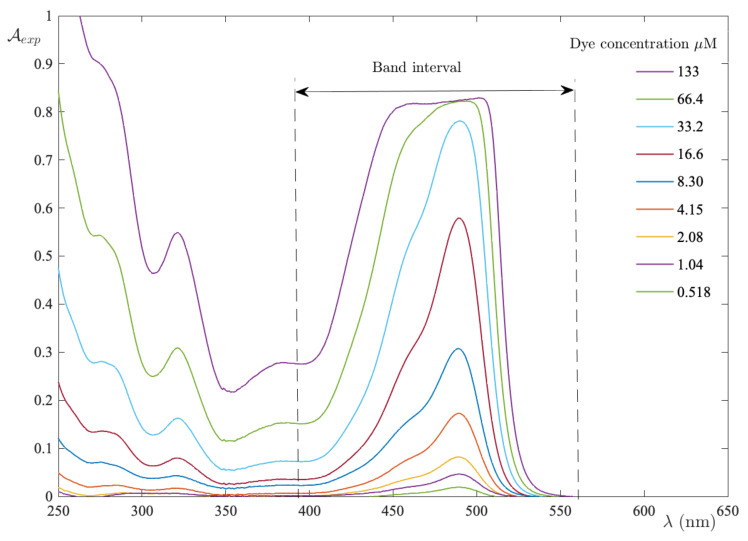
Absorption spectra recorded in the FS−UPW binary system as the dye concentration varies. The band interval indicates the range used to calculate the integral absorbance.

**Figure 2:**
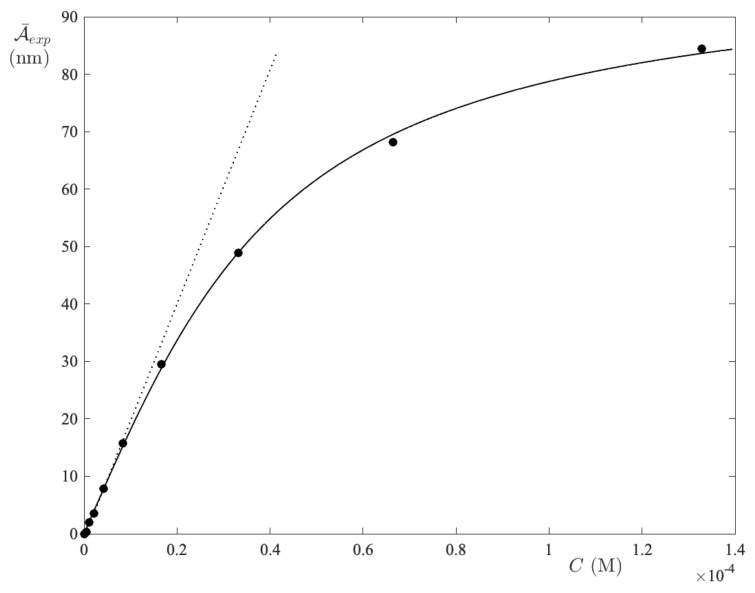
Integral absorbance vs. FS concentration in UPW. The limiting slope determines the absorption coefficient of the monomer form. The curve is a guide for the eyes.

All of the absorption spectra of FS-UPW solutions are collected in Figure 1, where one can be seen as a characteristic peak centered at 486 nm. As the dye concentration increases from zero to 133 μM, the peak remains substantially centered at the same wavelength, although the adsorption band tends to widen as a result of the strong interactions in solution [32]. In the band interval [390,552] nm, for each spectrum the integral absorbance was calculated and plotted as a function of the dye concentration (Figure 2).

**Figure 3:**
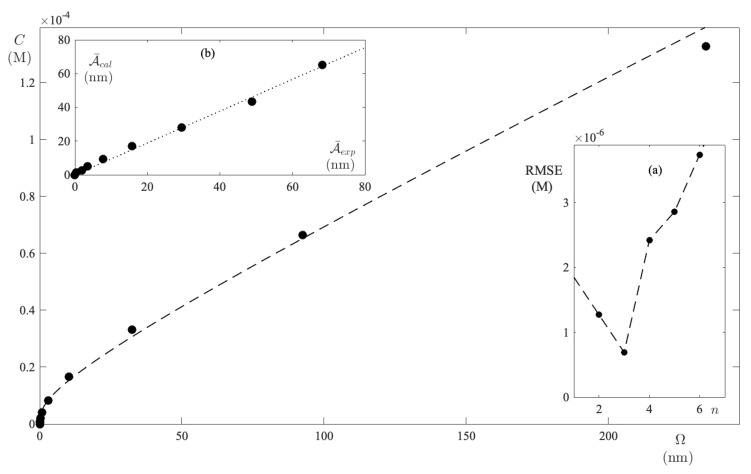
FS molar concentration in UPW as a function of excess absorbance. The dashed curve is the result of fitting to the model equation with n=3. Panel (**a**) displays the RMSE for each test performed at different *n*. Panel (**b**) shows the strong correlation between the integral absorbance when recalculated with the new parameters, as well as the experimental one.

The resulting curve, ${\bar{A}}_{exp}\left(C\right)$, plotted as function of FS concentration in Figure 2, exhibits a downward concavity, thereby entailing the fact that ${\bar{\epsilon }}_{1}>{\bar{\epsilon }}_{n}$. Moreover, a direct calculation of the initial slope (the dotted line in Figure 2) from the experimental curve provides ${\bar{\epsilon }}_{1}=0.241$ M${}^{-1}$. Once the monomer absorptivity is established, the fitting of data sets to Equation (14) was performed for different *n* values, as shown in Figure 3. The reliability of the individual steps is displayed in panel (a), where the RMSE vs. *n* exhibits a net minimum for n=3. Thus, the model predicts that fluorescein in an aqueous solution reaches the minimum of free energy in the trimer form. At the minimum, one extracts ${\bar{\epsilon }}_{3}=0.027$ M${}^{-1}$ and $K_{3}=1.4\times 10^{9}$. In addition. the values are in agreement with the results presented by Casalini et al. [33] in a diffusion study of sodium fluorescein in water.

Accordingly, the algebraic equation in respect of a reliability test assumes a cubic form

$$\left(4.2\times 10^{9}\cdot C^{2}\right)\xi^{3}+\xi -1=0$$

This equation was solved for each value of *C*. Then, the set of solutions $\left(C_{1},C_{3}\right)$ is exploited in order to compute ${\bar{A}}_{calc}$. The comparison between the calculated and experimental values was made by plotting ${\bar{A}}_{calc}$ vs. ${\bar{A}}_{exp}$. Moreover, the results displayed in panel (b) of Figure 3 indicate that ${\bar{A}}_{calc}$ = 0.943 ${\bar{A}}_{exp}$, i.e., the calculated integral absorbance differs from the experimental one by no more than 6%.

#### 2.2.2. Anionic Dye in Organic Solution

**Figure 4:**
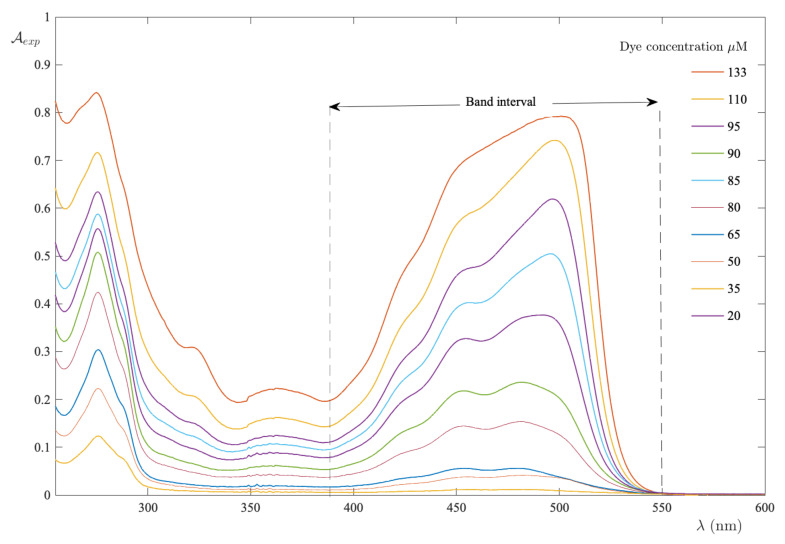
Absorption spectra recorded in the FS–ACN binary system as the dye concentration varies. Band interval indicates the range used to calculate the integral absorbance.

**Figure 5:**
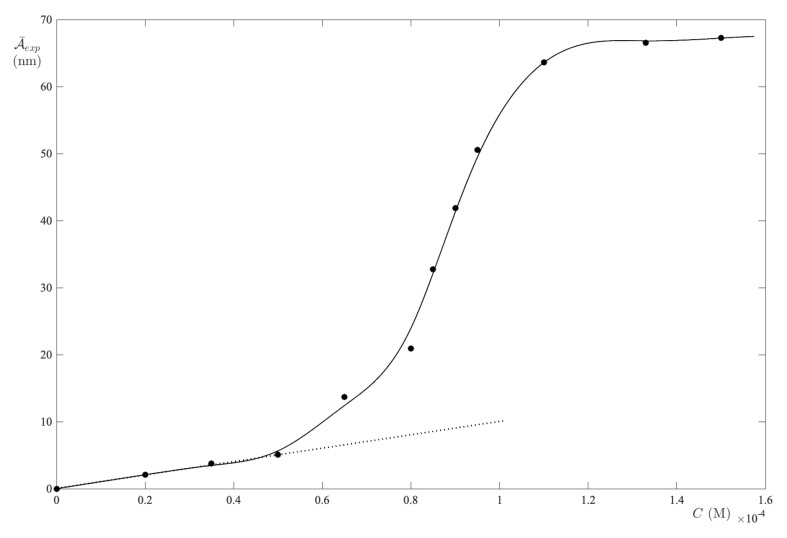
Integral absorbance vs. FS concentration in ACN. The limiting slope determines the absorption coefficient of the monomer form. The curve is a guide for the eyes.

The absorption spectra of two-anionic fluorescein, which are recorded in ACN solutions, are collected in Figure 4. As is immediately seen, the absorption peak is always centered at 486 nm, although the secondary peaks are also present here. Unlike in water, the curve ${\bar{A}}_{exp}\left(C\right)$ (see Figure 5) now exhibits an initially upward concavity, i.e., ${\bar{\epsilon }}_{1}<{\bar{\epsilon }}_{n}$. Indeed, the slope in the origin is very small (i.e., the dotted line in Figure 5). Since the type of dye and its concentration are the same as in UPW, one infers that the dye–dye interactions are solvent-mediated. Furthermore, the integration of the spectra was always carried out in the band interval [390,552] nm.

**Figure 6:**
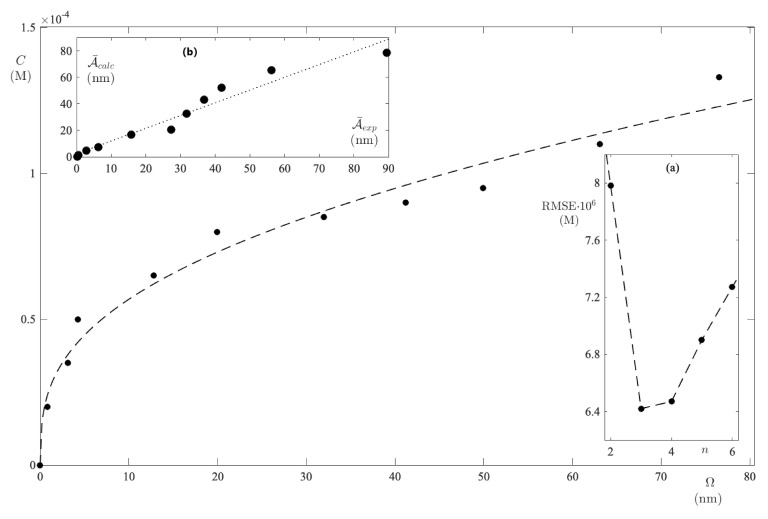
FS molar concentration in ACN as a function of excess absorbance. The dashed curve is the result of fitting to the model equation with n=3. Panel (**a**) displays the RMSE for each test performed at a different *n*. Panel (**b**) shows the strong correlation between the integral absorbance that is recalculated with the new parameters, as well as the experimental one.

When repeating the calculations, in respect of the UPW–FS solutions, the function C(Ω), which is reproduced in Figure 6, is found. In addition, the parameters ${\bar{\epsilon }}_{3}=0.389$ M${}^{-1}$ and $K_{3}=6.3\times 10^{6}$ are extracted. The RMSE vs. *n* plot displayed in panel (a) of Figure 6 shows that, even in ACN, the minimum occurs when n=3. However, it should be noted that the RMSE values for n=3 and n=4 differ slightly. In any case, the calculated integral absorbance for n=3 provides ${\bar{A}}_{calc}$ = 0.97${\bar{A}}_{exp}$ (as detailed in panel (b) of Figure 6).

In respect of this, the results appear to indicate that the number of molecules involved in the self-assembly process depends on the geometry and chemical structure of the dye molecule.

#### 2.2.3. Cationic Dye in Aqueous Solution

Unlike FS, MB can be dissociated into positively charged ions in an aqueous solution. Since MB is a Food and Drug Administration-approved medicine with a long history, the safety of MB usage has been thoroughly evaluated. Recently, this dye has also been employed in order to explore aging-related conditions. Furthermore, it can also help us understand the aging process [26].

**Figure 7:**
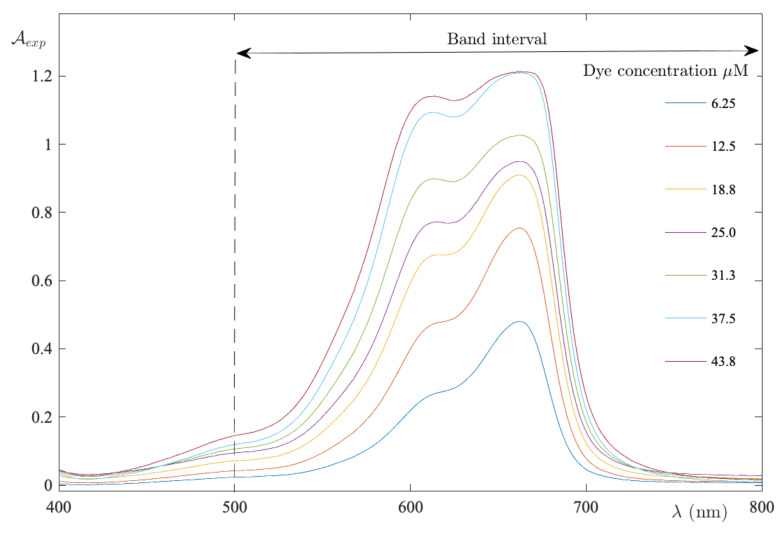
Absorption spectra recorded in the MB–UPW binary system as the dye concentration varies. The band interval indicates the range used to calculate the integral absorbance.

**Figure 8:**
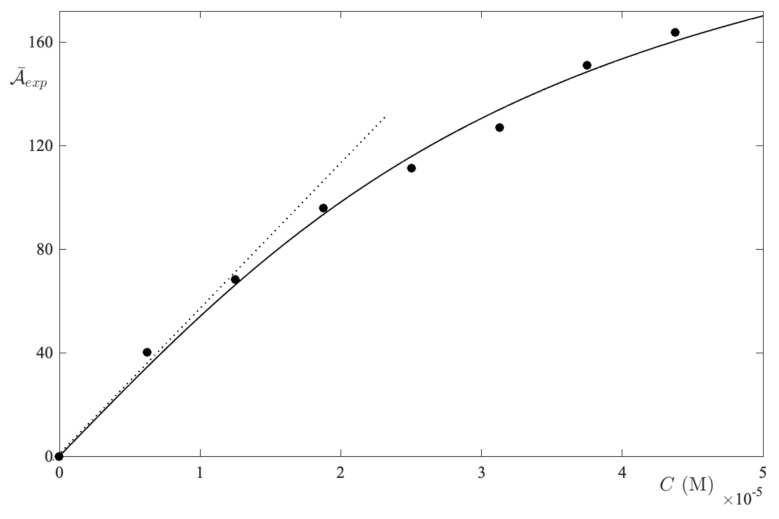
The integral absorbance vs. the MB concentration in UPW. The limiting slope determines the absorption coefficient of the monomer form. The curve is a guide for the eyes.

In the present work, we have measured the visible light absorption spectra of a large number of aqueous solutions in respect of MB in the concentration range of 6–44 μM. As one can see in Figure 7, the absorption spectra are composed of two bands, whereby the first is with the maximum at 661 nm, which is most prominent in dilute solutions. Whereas the second, with a maximum at 615 nm, is stronger in the concentrated solutions. Moreover, the integral absorbance was calculated in the 500–800 nm band interval. Further, the curve ${\bar{A}}_{exp}\left(C\right)$ exhibits a downward curvature, such that the difference between the experimental integral absorbance and the reference is negative throughout the band interval (Figure 8).

**Figure 9:**
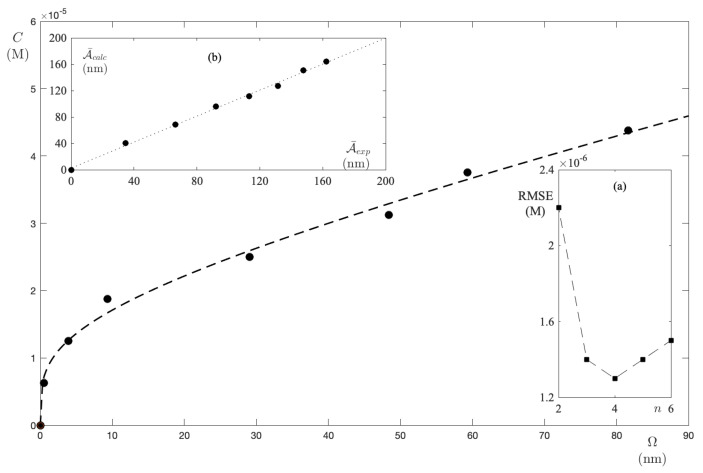
MB molar concentration in UPW as a function of excess absorbance. The dashed curve is the result of fitting to the model equation with n=4. Panel (**a**) displays the RMSE for each test performed at a different *n*. Panel (**b**) shows the strong correlation between the integral absorbance when it is recalculated with the new parameters and the experimental one.

As shown in Figure 9, the theoretical curve fits well with the experimental results. Furthermore, the RMSE reaches its minimum value for n=4 (Figure 9 and panel (a)), when $k_{4}=\left(1.15\pm 0.07\right)\times 10^{13}$.

As such, the model results appear to indicate that the predominant species at equilibrium is found in the tetramer. With these results, the resolution of Equation (16) provides the results displayed in panel (b) of Figure 9, i.e., ${\bar{A}}_{calc}=0.98{\bar{A}}_{exp}$. This result deserves a few words of comment. Indeed, in respect of studies regarding the self-aggregation of MB in water, the existence trimers or higher order n-mers have long been assumed [34,35,36]. Furthermore, certain studies have established the threshold of the trimers and n-mers’ formation, when at room temperature, is in the range 10–50 μM [37,38]. Since our results were obtained in the concentration range of 6–44 μM, we can infer that the conclusions reached with the excess absorbance are in agreement with the results that were obtained with much more complex numerical techniques [39]. Furthermore, the analysis of the MB spectra in UPW showed that this dye tends to form tetramers in an aqueous solution. This result appears to corroborate the recent data obtained through specific and complex numerical models.

## 3. Materials and Methods

### 3.1. Materials

Fluorescein sodium salt (FS), methylene blue (MB), as well as the bioreagents and acetonitrile (ACN) (gradient grade, purity 99.9%) were all purchased by Sigma Aldrich and used without further purification. The water used for preparing solutions was ultra pure water (UPW), produced by the Milli-Q system. The pH was measured on the freshly prepared solutions and then again at the end of the absorption measurements. The deviations were all contained within the range of 6.9–7.2.

### 3.2. Methods

#### 3.2.1. Preparation of the Samples

Stock solutions were prepared by dissolving the dye (5 mg) in 10 mL of UPW or ACN. Each sample was then obtained by diluting the corresponding stock solution up to the desired concentration. In particular, the aqueous solutions were prepared in the concentration range of 0.518–133 μM, while the organic solutions were in the range 20–133 μM. Furthermore, the stock solution of MB was prepared by dissolving 0.140 g in 10 mL. All aqueous MB solutions were in the range 6–44 μM.

#### 3.2.2. Absorption Measurements

Absorption measurements were performed by a Cary 100-Varian UV-Vis equipped with thermostatted cells. Liquid mixtures were placed in rectangular quartz cells of a 1 cm path length. Furthermore, the absorption spectra were recorded at (20.0 ± 0.5) ${}^{\circ }$C in the 250–650 nm wavelength region for the fluorescein-UPW binary system, and in the 250–600 nm wavelength region for the fluorescein-ACN binary system. For the MB–UPW systems, the wavelength region was 400–800 nm.

## 4. Conclusions

Although this approach may account for a number of experimental results, it is vulnerable to certain criticisms. For instance, the treatment detailed in this study does not apply to simultaneous equilibria involving several polymeric species (it is true, however, that if only two equilibria were involved then a plot such as that of Figure 3 could be helpful in bracketing the experimental curve; then, one could fit the data by the appropriate complex equilibrium expression). Another more specific problem is that there is little assurance that ${\bar{\epsilon }}_{1}$ and ${\bar{\epsilon }}_{n}$ are unchanged over the whole concentration range. A good reconstruction of the experimental data indicates that the changing concentration has almost no effect on the extinction coefficients. While it cannot be stated with certainty that ${\bar{\epsilon }}_{1}$ and ${\bar{\epsilon }}_{n}$ must also remain constant, it is probable that the change in ${\bar{\epsilon }}_{3}$ will be parallel (roughly) as that in ${\bar{\epsilon }}_{1}$, such that the overall effect will be reduced. Since it is not apparent how the treatment effect on $K_{n}$ should be treated, we have estimated the uncertainty of about 7–8% in our $K_{3}$ via three repeated tests.

Our results underline the fact that fluorescein undergoes self-aggregation in polar protic and aprotic solvents in order to form trimers. It is likely that the planarity of the molecular skeleton, as well as the electrostatic interactions with counter-ions and hydrogen bonding may be responsible for the aggregation phenomenon. Our treatment reveals that fluorescein, in polar protic solvent (i.e., water), favors the aggregation more than the aprotic solvent. This finding is consistent with the data for phenosafranin and safranin, which were implemented in protic and aprotic polar solvents [40]. Furthermore, the analysis of the absorption spectra of MB in UPW by excess absorbance showed that the predominant species in the solution is the tetramer. In the same concentration range investigated in this study, the trimeric and tetrameric species in aqueous solutions have been hypothesized for some time. Furthermore, it was only recently that it was demonstrated through complex calculation methods. Therefore, the simplicity of the proposed method could be an aid for understanding the associative equilibria of dyes, both in aqueous and organic solutions. 

## Abbreviations

The following abbreviations are used in this manuscript:

FS	Fluorescein sodium salt;
ACN	Acetonitrile;
UPW	Ultra pure water;
UV-Vis	Ultraviolet-visible;
MB	Methylene blue

## References

[1] Luo S., Zhang E., Su Y., Cheng T., Shi C. (2011). A review of NIR dyes in cancer targeting and imaging. Biomaterials.

[2] Turner A.P.F. (2000). Biosensors–sense and sensitivity. Science.

[3] Grätzel M. (2011). Photoelectrochemical cells. Materials For Sustainable Energy: A Collection of Peer-Reviewed Research and Review Articles from Nature Publishing Group.

[4] Rodrigues E.B., Penha F.M., Furlani B., Meyer C.H., Maia M., Farah M.E. (2008). Historical aspects and evolution of the application of vital dyes in vitreoretinal surgery and chromovitrectomy. Vital Dyes in Vitreoretinal Surgery.

[5] Zeppa L., Ambrosone L., Guerra G., Fortunato M., Costagliola C. (2014). Using canalography to visualize the in vivo aqueous humor outflow conventional pathway in humans. JAMA Ophthalmol..

[6] Philip R., Penzkofer A., Bäumler W., Szeimies R., Abels C. (1996). Absorption and fluorescence spectroscopic investigation of indocyanine green. J. Photochem. Photobiol. A Chem..

[7] Proulx S.T., Luciani P., Derzsi S., Rinderknecht M., Mumprecht V. (2010). Quantitative imaging of lymphatic function with liposomal indocyanine green. Cancer Res..

[8] Chapman M., Mullen M., Novoa-Ortega E., Alhasani M., Elman J.F., Euler W.B. (2016). Structural evolution of ultrathin films of rhodamine 6g on glass. J. Phys. Chem. C.

[9] Prausnitz J.M., Lichtenthaler R.N., De Azevedo E.G. (1998). Molecular Thermodynamics of Fluid-Phase Equilibria.

[10] Serra F., Terentjev E.M. (2008). Nonlinear dynamics of absorption and photobleaching of dyes. J. Chem. Phys..

[11] Huong A., Tay K.G., Ngu X. (2019). Towards skin tissue oxygen monitoring: An investigation of optimal visible spectral range and minimal spectral resolution. Univ. J. Electr. Electron. Eng..

[12] Ong P.E., Huong A.K.C., Ngu X.T.I., Mahmud F., Philimon S.P. (2019). Modified lambert beer for bilirubin concentration and blood oxygen saturation prediction. Int. J. Adv. Intell. Inform..

[13] Contini D., Martelli F., Zaccanti G. (1997). Photon migration through a turbid slab described by a model based on diffusion approximation. I. Theory. Appl. Opt..

[14] Hiraoka M., Firbank M., Essenpreis M., Cope M., Arridge S.R., Van Der Zee P., Delpy D.T. (1993). A Monte Carlo investigation of optical pathlength in inhomogeneous tissue and its application to near-infrared spectroscopy. Phys. Med. Biol..

[15] Karamavuş Y., Özkan M. (2019). Newborn jaundice determination by reflectance spectroscopy using multiple polynomial regression, neural network, and support vector regression. Biomed. Signal Process. Control.

[16] Hagfeldt A., Boschloo G., Kloo L., Petterson H. (2010). Dye-sensitized solar cells. Chem. Rev..

[17] Kim H., Schembri T., Bialas D., Stolte M., Würthner F. (2022). Slip-stacked J-aggregate materials for organic solar cells and photodetectors. Adv. Mater..

[18] Li Y., Wang Y., Bian C., Stejskal J., Zheng Y., Jing X. (2020). Azo dye aggregates and their roles in the morphology and conductivity of polypyrrole. Dyes Pigments.

[19] Hawe A., Rispens T., Herron J.N., Jiskoot W. (2011). Probing bis-ans binding sites of different affinity on aggregated igg by steady-state fluorescence, time-resolved fluorescence and isothermal titration calorimetry. J. Pharm. Sci..

[20] Duff D., Kirkwood D., Stevenson D., Jiskoot W. (1977). The behaviour of dyes in aqueous solutions. The influence of chemical structure on dye aggregation a polarographic study. J. Soc. Dye. Colour..

[21] Inglesby M., Zeronian S. (2001). Diffusion coefficients for direct dyes in aqueous and polar aprotic solvents by the nmr pulsed-field gradient technique. Dyes Pigments.

[22] Berlepsch V., Böttcher C. (2015). H-aggregates of an indocyanine cy5 dye: Transition from strong to weak molecular coupling. J. Phys. Chem. B.

[23] Di Nezza F., Guerra R., Costagliola C., Zeppa L., Ambrosone L., Bracewell D.G. (2016). Thermodynamic properties and photodegradation kinetics of indocyanine green in aqueous solution. Dyes Pigments.

[24] Oshinbolu S., Shah G., Finka G., Molloy M., Uden M. (2018). Evaluation of fluorescent dyes to measure protein aggregation within mammalian cell culture supernatants. J. Chem. Technol. Biotechnol..

[25] Di Nezza F., Zeppa L., Costagliola C., Bufalo G., Ambrosone L. (2019). A physicochemical study of ophthalmological vital dyes: From dimerization equilibrium in buffer solution to their liposomal dispersions. Dyes Pigments.

[26] Xue H., Thaivalappil A., Cao K. (2021). The Potentials of Methylene Blue as an Anti-Aging Drug. Cells.

[27] Courrier E., Renault D., Kaspi M., Marcon A., Lambert V., Garcin T., Chiambaretta F., Garhofer G., Thuret G., Gain P. (2018). Micro-instillation of fluorescein with an inoculation loop for ocular surface staining in dry eye syndrome. Acta Ophthalmol..

[28] Jalli P., Hellsted T.J., Immonen I. (1997). Early versus late staining of microaneurysms in fluorescein angiography. Retina.

[29] Romano M.R., Ilardi G., Ferrara M., Cennamo G., Parolini B., Mariotti C., Staibano S., Cennamo G. (2018). Macular peeling-induced retinal damage: Clinical and histopathological evaluation after using different dyes. Graefe Arch. Clin. Exp. Ophthalmol..

[30] Jaffe H.H., Orchin M. (1962). Theory and Applications of Ultraviolet Spectroscopy.

[31] Ambrosone L., Sartorio R., Vitagliano V. (1993). Density measurements in the ternary system poly (vinylidene fluoride)-water-N,N-dimethyl formamide at 20 ^∘^C. Fluid Phase Equilibria.

[32] DeSilva L.A., Pitigala P., Gaquere-Parker A., Landry R., Hasbun J.E., Martin V., Bandara T.M.W.J., Perera A.G.U. (2017). Broad absorption natural dye (Mondo-Grass berry) for dye sensitized solar cell. J. Mater. Sci. Mater. Electron..

[33] Casalini T., Salvalaglio M., Perale G., Masi M., Cavallotti C. (2011). Diffusion and aggregation of sodium fluorescein in aqueous solutions. J. Phys. Chem. B.

[34] Braswell E. (2005). Evidence for trimerization in aqueous solutions of methylene blue. J. Phys. Chem..

[35] Heger D., Jirkovsky J., Klan P. (2005). Aggregation of methylene blue in frozen aqueous solutions studied by absorption spectroscopy. J. Phys. Chem. A.

[36] Zhao Z., Malinowski E.R. (1999). Determination of the hydration of methylene blue aggregates and their dissociation constants using visible spectroscopy. Appl. Spectrosc..

[37] Hemmateenejad B., Absalan G., Hasanpour M. (2011). Application of multivariate curve resolution analysis for studying the thermodynamics of methylene blue aggregations in aqueous solutions. J. Iran. Chem. Soc..

[38] Klika Z., Čapková P., Horáková P., Valášková M., Malỳ P., Macháň R., Pospíšil M. (2007). Composition, structure, and luminescence of montmorillonites saturated with different aggregates of methylene blue. J. Colloid Interface Sci..

[39] Fernandez-Perez A., Marban G. (2020). Visible light spectroscopic analysis of methylene blue in water; what comes after dimer?. ACS Omega.

[40] Sarkar D., Das P., Girigoswami A., Chattopadhyay N. (2008). Spectroscopic characterization of phenazinium dye aggregates in water and acetonitrile media: Effect of methyl substitution on the aggregation phenomenon. J. Phys. Chem. A.

